# A Multifaceted Approach to the “Bare below the Elbow” Concept and Hand Hygiene Compliance among Healthcare Professionals—Multicenter Population-Based Study

**DOI:** 10.3390/ijerph20054435

**Published:** 2023-03-02

**Authors:** Emilia Szumska, Przemyslaw Czajkowski, Michal Zablocki, Dorota Rozkiewicz

**Affiliations:** 1 Medilab Sp. z o. o., Niedzwiedzia 60, 15-531 Bialystok, Poland; 2 Clinical Research Centre, Medical University of Bialystok, Jana Kilinskiego 1, 15-089 Bialystok, Poland; 3 Department of Pediatric Infectious Diseases, Medical University of Bialystok, Waszyngtona 17, 15-274 Bialystok, Poland

**Keywords:** bare below the elbow, BBE, disinfection, hand hygiene, antiseptics, infection prevention

## Abstract

Nosocomial infections remain an important issue for patient safety concerns. Since hospital infections are mainly connected with healthcare professionals’ routines, an increase in hand hygiene effectiveness through compliance with the “bare below the elbow” (BBE) concept could reduce the number of nosocomial infections. Therefore, this study aims to evaluate hand hygiene and to investigate healthcare professionals’ compliance with the BBE concept. We performed our study on a group of 7544 hospital professionals involved in patient care. During the national preventive action, questionnaires, demographic data, and hand hygiene preparations were recorded. Hand disinfection was verified by *COUCOU BOX*, containing a UV camera. We noted that 3932 (52.1%) persons complied with the BBE rules. Nurses and non-medical personnel were significantly more often classified as BBE rather than non-BBE (2025; 53.3% vs. 1776; 46.7%, respectively, *p* = 0.001 and 1220; 53.7% vs. 1057; 46.3%, *p* = 0.006). Different proportions were demonstrated for the groups of physicians—non-BBE (783; 53.3%) compared to BBE (687; 46.7%) (*p* = 0.041). Healthcare workers from the BBE group statistically more often disinfected their hands correctly (2875/3932; 73.1%) compared to the non-BBE group (2004/3612; 55.5%) (*p* < 0.0001). This study demonstrates the positive impact of compliance with the BBE concept on effective hand disinfection and patient safety. Therefore, education and infection-prevention actions should be popularized to improve the BBE policy’s effectiveness as well.

## 1. Introduction

The elimination of nosocomial infections is practically an unrealistic process, although the knowledge concerning this subject and the prevention protocols is considerable. It should be emphasized that the epidemiological situation of a hospital not only affects the level of medical assistance and patient safety numbers but also prolongs hospitalization and increases treatment costs. Considering the prevention of hospital infections and the concept of hand hygiene, promotional campaigns should be performed. Only nationwide infection-control actions can provide a significant reduction in healthcare-associated infections. The creation of restrictive guidelines for improving hand hygiene and the implementation of appropriate actions affecting the correctness of the hand disinfection procedure may be a turning point in reducing nosocomial infections.

Adherence to the “bare below the elbow” (BBE) concept also has a significant impact on the correctness of the hand disinfection procedure and uniform requirements (short-sleeved clothing) among medical and non-medical personnel in medical facilities. Moreover, according to BBE policy, medical personnel should take care of the appearance of their nails, and it is forbidden to wear jewelry and ties [1,2]. Taking the abovementioned factors into consideration, it can be observed that there is a need to spread the BBE phrase among healthcare professionals and implement it as a mandatory practice to improve patient safety, as is performed in the United Kingdom [1], other European countries, the USA, and Canada.

The next key of the multimodal hand hygiene improvement strategy is the education of healthcare workers [3,4]. More focus should be set on the proper technique procedure of cleaning hands and, moreover, adequate time for washing. The assumption of the WHO was that hand hygiene promotion activities, which are a national priority, would lead to sustainable development on a global scale. It has been shown that multifaceted-wide strategies should be implemented as a routine rather than single-element institution-wide strategies [5,6]. In fact, among healthcare practitioners, audiovisual media results in higher adherence than traditional teaching methods [7].

According to WHO, the prevalence of healthcare-associated infections is estimated to be in the range of 3.5–19.1%, dependent on the countries’ income (low–high), even considering the under-reporting of healthcare-associated infections from many countries [8]. Contaminated hands of healthcare practitioners are one of the main sources of nosocomial infections; therefore, strategies to keep hands clean have always been of utmost importance [9]. Nevertheless, hand hygiene might appear as a common and simple procedure, but compliance in healthcare settings has always been far from perfect worldwide [10,11]. Unfortunately, the estimated hand hygiene compliance rate has been reported as 40% in high-income countries and less than 20% in low-income countries [8,12].

Although hand hygiene is very important in the prevention of healthcare-associated infections, there remains no global unity among medical professionals concerning the BBE concept and its influence on the effectiveness of hand hygiene. The aim of this study is to evaluate hand hygiene and investigate healthcare professionals’ compliance with the BBE concept. This study is the first systematic evaluation of hand disinfection techniques among medical and non-medical personnel of Polish origin from a multicenter aspect.

## 2. Materials and Methods

### 2.1. Participants of the Study Cohort

The study group comprised 7544 adults of Polish origin, recruited from 123 Polish healthcare facilities and deployed in 12 regions of Poland, as previously described [13]. All observations were performed among persons working at different parts of the hospital: the medical clinic, long-term care facilities (LTCFs), and administration offices. All of the procedures and observations were performed on medical (physicians and nurses) and non-medical personnel (administration specialists, radiologic technologists, pharmacists, laboratory staff, physiotherapists, and cleaning and food services). Compliance with hand hygiene procedures and the BBE concept was recorded in the included study cohort.

### 2.2. Study Examination

The study procedures were performed on the participants during their routine work in healthcare facilities: hospital departments, medical clinics, and long-term-care facilities (LTCFs). A series of training, dedicated to hand hygiene, was conducted as a part of an educational campaign, “Close the door to hospital infections”, combined with a multimedia presentation. The educational and preventive actions, organized by the firm Medilab Sp. z o. o. and the Scientists of the Medical University of Bialystok under the auspices of Polish Scientific Associations, were addressed to healthcare professionals. During the campaign, the volunteers participated in a theoretical demonstration and training of hand hygiene and disinfection techniques according to the Ayliffe hand hygiene technique recommended by the WHO [14].

Prior to the start of the assessment of hand disinfection techniques, all volunteers were asked, inter alia, whether they were, at that moment in time, wearing workplace/medical clothes, usually worn in the patient zone. Subsequently, participants disinfected their hands with one dose (3 mL) of fluorescent *Aniosgel 85 NPC* (Anios Laboratoires, Lezennes, France). We used, in our research, two outside observers/investigators who were well-trained and used a validated protocol of the study. Additionally, the observers had no personal relationships with the participants, and no exception to the procedure protocol was allowed. The external investigator noted the hand preparation conducted for the procedure and disinfection itself on an anonymous form. In another anonymous questionnaire, an external investigator recorded the sex, profession, detailed place of work, job seniority, dominant hand, and level of preparation of hands for disinfection, including risk factors (e.g., lack or presence of artificial/polished nails; long nails that protrude beyond the surface of the skin; irritated skin; presence of watches, bracelets, rings, and long sleeves that were not rolled up). The verification of the correctness of hand and wrist disinfection was examined by the device connected to a UV camera—*COUCOU BOX* (Anios Laboratories, Lezennes, France). With the use of a computer screen, the captured images of the hands and wrists were assessed. Hand disinfection was considered effective and adequate when the hand skin area on which the bright UV light was reflected was at least 94% of the whole hand’s surface. The dorsal and inner sides of the left and right hands were assessed for each volunteer. The palm area was divided into zones, i.e., the fingertip and thumb area and the lateral, central, and dorsal sides of the hands. All of the zones of both hands accounted for 100% of the hand surface.

### 2.3. Ethics Statement

The research methods followed the ethical standards of the Declaration of Helsinki. The Ethics Committee of the Medical University of Bialystok, Poland, approved the study protocol (R-I-002/180/2017). Written informed consent was provided by all voluntary participants prior to the start of the procedure. All volunteers were informed about the purpose of the study. Participants had the right to withdraw from the study at any time.

### 2.4. Statistical Analysis

The numerical data were presented as percentages and summarized with the number of observations (n). Groups were compared with Mann–Whitney and one-way analysis of variance (ANOVA) Kruskal–Wallis tests, where appropriate. The chi-squared test was used to compare the independent proportions of a normal distribution. The relative probability of inadequate hand hygiene was determined using an odds ratio (OR) with a 95% confidence interval (95% CI). All calculations were performed in STATA version 10.0 software (StataCorp, College Station, TX, USA). The statistical significance level was set at <0.05.

## 3. Results

The data obtained from 7544 participants (84% female and 16% male) were included in the analysis, as previously described [13]. Of the study cohort, medical workers comprised 19.5% physicians, 50.4% nurses, and 30.1% other personnel. The others included non-medical staff: 405 administration specialists (17.8%), 191 radiologic technologists (8.4%), 108 pharmacists (4.8%), 101 laboratory staff (4.4%), 227 physiotherapists (10.0%), 1016 cleaning service staff (44.7%), and 225 food service staff (9.9%). Among the study population, 6772 volunteers participated in the study for the first time and 772 for the second time. The general characteristic of the studied population has been previously presented [13].

### Assessment of Compliance with the BBE Concept

Among 7544 participants, 3932 (52.1%) of them applied the BBE concept (BBE group) (Table 1), whereas out of 3612 people (47.9%), there were BBE concept deviations (long nails, artificial/painted nails, rings, watches, bracelets, long sleeves); these people were defined as a non-BBE group. Nurses and non-medical personnel were more frequently present in the BBE group than in the non-BBE group (53.3% vs. 46.7%, respectively, *p* = 0.001 and 53.7% vs. 46.3%, *p* = 0.006). Inverse proportions were observed in the group of physicians: non-BBE (783; 53.3%) compared to BBE (687; 46.7%) (*p* = 0.041).

We noted that among the BBE group, non-medical personnel (1220; 53.7%) were more frequently present than medical personnel (2712; 51.5%) (*p* < 0.0001). The statistical analysis showed that among non-medical personnel, including their profession, most of the cases were present in the BBE group; however, only in the case of physiotherapists (67.8% vs. 32.2%) (*p* = 0.0001) and laboratory workers (60.4% vs. 39.6%) (*p* = 0.0031) were significant differences observed. Statistically significant differences were not observed between the remaining non-medical personnel: cleaning staff—*p* = 0.2141; food service—*p* = 0.7820; administration specialists—*p* = 0.0578, and pharmacists—*p* = 0.4142 (Table 1).

**Figure 1:**
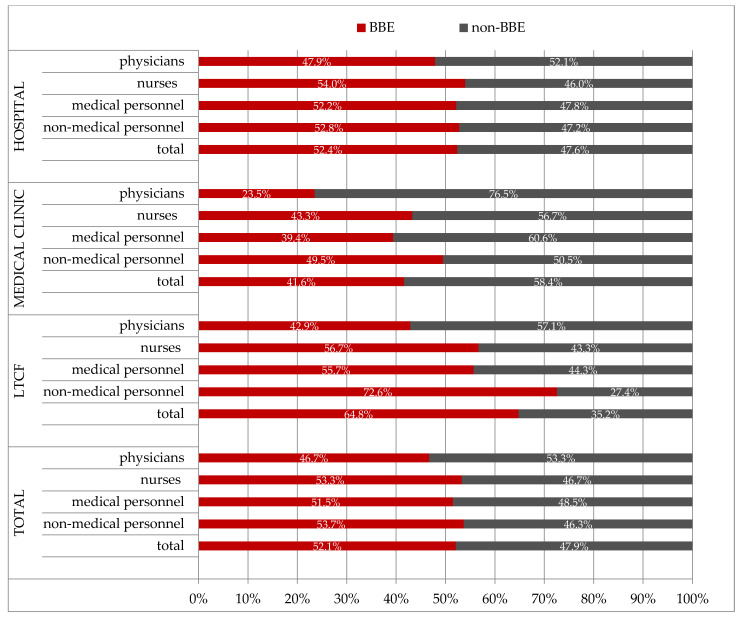
The frequency of adherence to the BBE policy depends on the person’s profession and place of work (LTCFs—long-term-care facilities).

We observed that among the 6896 volunteers employed in the hospital, 3614 (52.4%) were classified in the BBE group (Figure 1). This frequency was significantly higher compared to the personnel employed in medical clinics (182/438; 41.6%) (*p* < 0.0001) and significantly lower compared to the personnel employed in LTCFs (136/210; 64.8%, *p* < 0.0001).

Moreover, we did not observe significant differences in the compliance with the BBE concept between medical and non-medical personnel employed in the hospital (2523/4831; 52.2% vs. 1091/2065; 52.8%, respectively, *p* = 0.6435) and clinic (135/343; 39.4% vs. 47/95; 49.5%, respectively, *p* = 0.0767). In LTCFs, non-medical personnel (82/113; 72.6%) were more often present in the BBE group compared to medical personnel (54/97; 55.7%) (*p* = 0.0106). We noted that only every fourth physician (16/68; 23.5%) employed in the clinic complied with the BBE concept (Figure 1).

Furthermore, we assessed the detailed analysis of compliance with the BBE concept among people employed in the hospital, depending on the workplace/department (Table 2). In the surgical departments, most of the employees were from the BBE group (1297/2208; 58.7%) rather than the non-BBE group (911/2208; 41.3%) (*p* < 0.0001). The same relationship was observed among ICU staff (215/399; 53.9% and 184/399; 46.1%, respectively) (*p* = 0.0282). In the general departments, half of them were in the BBE (1471/2971; 49.5%) and non-BBE groups (1500/2971; 50.5%) (*p* = 0.4518). Only ED staff were significantly more likely to belong to the non-BBE group than to the BBE group (228/391; 58.3% vs. 163/391; 41.7%, respectively) (*p* < 0.0001).

We observed that the personnel of the surgical wards most regularly complied with the BBE concept (58.7%) compared to the personnel from the general departments (49.5%), ED (41.7%), or others (50.5%) (*p* < 0.0001).

The vast majority (5179/6896; 75.1%) of study participants were employed in surgical or general departments; therefore, we compared the participants from different professional groups, in particular, the location of work. It was shown that both physicians and nurses from surgical departments significantly more often complied with the BBE concept compared to those employed in general departments (respectively, 334/577; 57.9% and 750/1264; 59,3% vs. 224/589; 38.0% and 791/1 514; 52.2%; *p* < 0.0001 and *p* = 0.0002). Moreover, we observed that among non-medical personnel, in particular, participants from the surgical departments were more often in the BBE group (213/367; 58.0%) compared to the general departments (456/868; 52.5%); however, this difference was not statistically significant (*p* = 0.0761) (Table 2).

**Figure 2:**
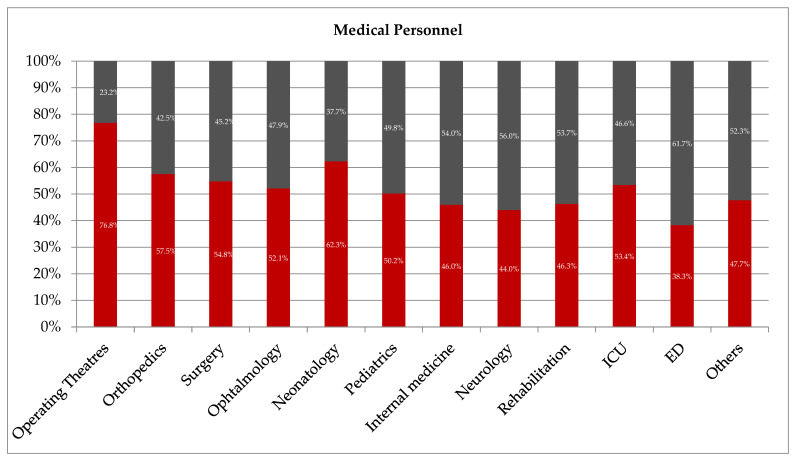
The frequency of compliance with the BBE policy among hospital medical personnel (other—administration, radiology, laboratory, pharmacy; ICU—intensive care unit; ED—emergency department). Black color—non-BBE group; red color—BBE group.

We noted that only 23.2% of medical personnel employed in the operating theatre were in the non-BBE group, and this observation presented a significantly lower frequency value compared to physicians and nurses employed in other departments (*p* < 0.05) (Figure 2). In internal medicine, neurology, and rehabilitation departments, the majority of medical personnel were significantly more often present in the non-BBE than in the BBE group (54.0% vs. 46.0%; *p* = 0.0001, 56.0% vs. 44.0%; *p* = 0.0291 and 53.7% vs. 46.3%; *p* = 0.1646, respectively).

**Figure 3:**
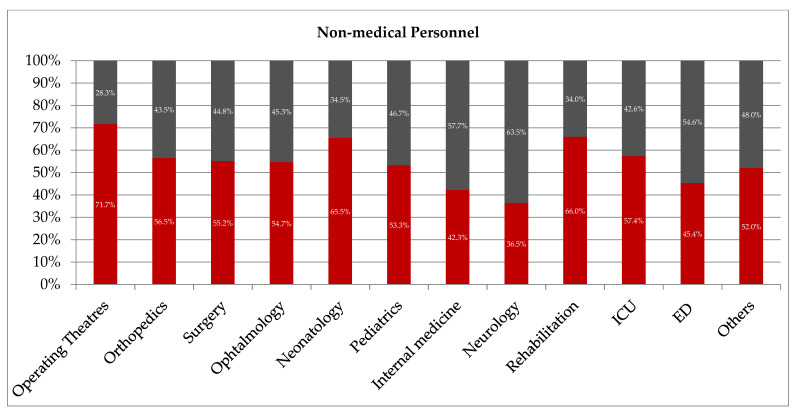
The frequency of adherence to the BBE policy depends on the personnel’s profession and place of work (other—administration, radiology, laboratory, pharmacy; ICU—intensive care unit; ED—emergency department). Black color—non-BBE group; red color—BBE group.

Similar to the results presented above, the non-medical personnel employed in internal medicine and neurology departments also did not comply with the requirements of the BBE concept (non-BBE 57.7% vs. BBE 42.3%; *p* < 0.0001 and 63.5% vs. 36.5%; *p* = 0.0025) (Figure 3).

Additionally, the odds ratio (OR) with a 95% confidence interval (95%CI) was calculated to estimate the BBE concept and hand hygiene compliance among healthcare professionals. The chance of BBE concept compliance among participants increased when they were employed in operating theatres—OR = 3.1 95% CI (2.48–3.89), surgical departments—OR = 1.46 95% CI (1.32–1.61), neonatology—OR = 1.62 95% CI (1.30–2.02), and rehabilitation—OR = 1.29 95% CI (1.06–1, 57). Furthermore, the chance of adherence to the BBE concept among participants decreased when they were employed in the ICU—OR = 0.64 95% CI (0.52–0.79), ED—OR = 0.64 95% CI (0.52–0.79), neurology department—OR = 0.66 95% CI (0.50–0.86), general departments—OR = 0.84 95% CI (0.77–0.92), and pediatrics—OR = 0.94; 95% CI (0.74–1.20).

The analysis of job seniority showed that medical personnel with ≤10 years of work experience were more likely to be in the BBE group (1596/3117; 51.2%) than in the non-BBE group (1521/3117; 48.8%) (*p* = 0.0575). A similar relationship was demonstrated for participants with over 10 years of work experience (1116/2154; 51.8% vs. 1038/2154; 48.2%), and it was a statistically significant difference (*p* = 0.0175). Moreover, in the group of physicians with ≤10 and more than 10 years of work experience, we noted that non-BBE participants dominated (respectively 438/911; 48.1% vs. 473/911; 51.9% *p* = 0.1010 and 249/559; 44.5% vs. 310/559; 55.5% *p* = 0.0003). Among non-medical personnel with ≤10 years and longer, significantly more often were participants classified in the BBE than the non-BBE group (710/1344; 52.8% vs. 634/1344; 47.2% *p* = 0.0034 and 510/929; 54.9% vs. 419/929; 45.1% *p* < 0.0001). When we analyzed and compared the BBE group of non-medical personnel, there was no significant difference in job seniority (54.9% vs. 52.8%) (*p* = 0.3305).

Furthermore, in the subsequent two tables, we provide a detailed description of employees who adhered to the BBE concept (BBE group) (Table 3) and those who did not comply with this concept (non-BBE group) (Table 4).

Regarding gender, no statistically significant differences were observed between the BBE and non-BBE groups among medical and non-medical personnel, except for the professional group of physicians. Among physicians, men were significantly more often in the BBE (59.4%) group compared to women (40.6%) (*p* < 0.0001).

Physicians from the BBE group working in hospitals were statistically significantly more often employed in surgical departments (50.0%) than in general departments (33.5%) (*p* < 0.0001). Such a relationship was not demonstrated for the group of nurses (BBE 40.4% vs. 42.6%; *p* = 0.1720). Hospital personnel from the BBE group significantly more often were employed in the primary reference hospital (56.4%) compared to the secondary (24.9%) or tertiary levels (18.7%) (*p* < 0.0001) of the healthcare referral system.

**Figure 4:**
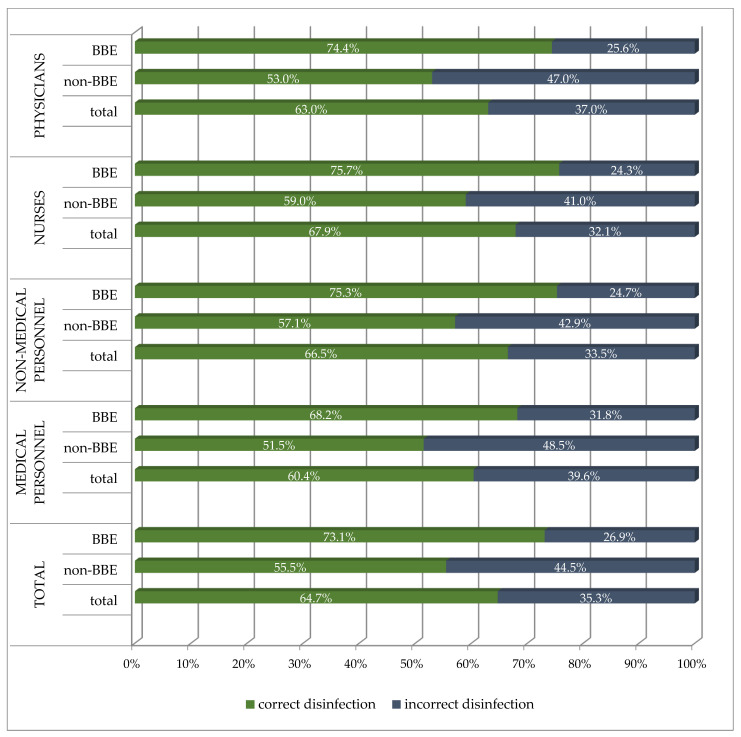
The frequency of hand disinfection in various professional groups, depending on the adherence to BBE compliance.

In the subsequent stage, the impact of compliance with the BBE concept on the correctness of hand disinfection was assessed (Table 5 and Figure 4). Among the 7544 employees, 4879 (64.7%) properly disinfected their hands. In this group, this statistically significantly occurred more often with people from the BBE group (2875/4879; 58.9%) compared to the non-BBE group (2004/4879; 41.1%) (*p* < 0.0001).

In the group of 3932 people who adhered to the BBE concept, 2875 (73.1%) correctly disinfected their hands, compared to 55.5% (2004/3612) from the non-BBE group, and it was a statistically significant difference (*p* < 0.0001). This correlation applied to all professional groups (Table 5 and Figure 4).

## 4. Discussion

To the best of our knowledge, this is one of the largest studies that represent the systematic evaluation of the BBE concept and hand hygiene compliance among medical and non-medical personnel from a multicenter aspect. Even though the recommendations of the BBE concept have been accepted and implemented by the National Health Service (NHS) in England [1], other European countries, the USA, and Canada, there remains no global consensus on the impact on the effectiveness of hand disinfection compliance. Therefore, there is a greater need for multicenter studies to evaluate the impact of the BBE concept on the correctness of hand disinfection.

Some of the studies have shown the lack of a significant effect of the abovementioned guidelines on the number of microbes colonizing hands and on the degree of their reduction after performing the hand hygiene procedure [15,16,17]. Burger et al. conducted studies in which they assessed the effect of clothing on the number of microbes present on the hands [16]. They showed no statistically significant difference in the number of bacterial colonies of pathogenic microorganisms between the control (non-BBE) and examined (BBE) groups. There was also no significant influence of jewelry on the diversity of bacteria. After performing the hand hygiene procedure, bacterial cells were reduced, while compliance with BBE recommendations did not contribute to a significant reduction in the number of bacteria. It turned out that the microbial count on the sleeves of protective clothing was comparable to the microbial count on the wrists.

Hand hygiene guidelines dedicated to medical staff only to some extent overlapped with the clothing policy. Moreover, according to the Centers for Disease Control and Prevention, it is not recommended that rings or wedding rings be worn by healthcare professionals, especially in cases of invasive procedures [18]. On the other hand, the WHO has more liberal rules and recommends that only rings, wedding rings, watches, and bracelets should be removed prior to proceeding with the surgical preparation of the hands. In other situations, the WHO allows the wearing of rings for religious and cultural reasons [14]. In the United Kingdom, since 2007, the NHS has strictly banned healthcare professionals from wearing jewelry and watches in the workplace [1]. The abovementioned inaccuracies and lack of uniformity led us to perform our previous study [13] concerning hand hygiene deviations (long nails, artificial/painted nails, rings, watches, bracelets, and long sleeves) and, furthermore, to broaden the knowledge of BBE compliance at present.

Our study showed that the group of physicians was significantly less frequently classified in the BBE group compared to nurses and, surprisingly non-medical personnel.

Many studies have shown that as the hierarchy of medical personnel evolves, the percentage of properly performed hand hygiene procedures decreases [14,19,20]. Oftentimes, physicians are less successful in following handwashing and disinfecting procedures than nurses. The question of the correlation between the omission of hand hygiene procedures and the importance of other activities performed while caring for patients has begun to be considered in the literature. There are assumptions that the behavior of skipping hand hygiene procedures is psychological and poses a severe ethical problem [20,21,22,23,24].

Moreover, Hautemaniere et al. showed that experienced nurses were more effective in the hand disinfection procedure compared to other professional groups (including physicians, cleaning staff, and radiologic technologists). Similarly, these results support our study and show that profession is an important factor influencing the correctness of the hand hygiene procedure [25].

Skodova et al. applied a methodology similar to that presented in our study, assessing the correctness of the hand disinfection procedure performed under a UV lamp. The study involved 133 doctors (18.87%), 241 nurses (34.18%), 214 nursing assistants (30.35%), and 117 representatives of other professions (16.61%). The nursing staff was the group that achieved the highest hand hygiene score throughout the entire study period. Only 67 employees (9.5% of respondents) achieved a 100% disinfection score, i.e., all areas of the hands were properly covered with an alcohol-based disinfectant [26].

Diversified compliance with hand hygiene, depending on the occupational group, was shown in the analysis conducted by Randle et al. [27]. With 24 h monitoring, the following hand hygiene compliance rates were obtained: physicians—47%, nurses—75%, auxiliary nurses—59%, patients—56%, and visitors—57%. The probability of observing good hand hygiene was higher for all occupational groups compared to the medical group. In the case of nurses, the chance of observing good hand hygiene was more than twice as high as in the case of physicians. It is a worrying fact that compliance with hand hygiene in the group of physicians was low, even compared to patients and visitors.

In another study conducted by Pittet et al. [28], the mean hand hygiene compliance rate obtained by a direct follow-up study in 2834 indications was 48%. Again, a higher level of compliance with hand hygiene procedures was recorded among nurses than in other professional groups. It was shown that the rate of non-compliance with hand hygiene was highest among physicians, the nursing staff, and other personnel [28]. In addition, a multivariate analysis showed that hand hygiene non-compliance with indications was the lowest during weekends and that non-compliance was higher in the intensive care unit than in the wards of internal medicine, as well as in patients requiring numerous procedures. The researchers concluded that targeted educational programs were appropriate and suggested that staff shortages could reduce the quality of patient care.

Interesting results were obtained during another observation performed at the University Hospital in Geneva [19]. The mean hand hygiene compliance rate was 57%, and it differed depending on the medical specialty, i.e., general practitioners—87% and anesthesiologists—23%. In addition, it was noted that hand hygiene compliance among physicians was greater (61%) when they were aware that they were being watched compared to when they were not aware of it. Observations conducted in the Geneva hospital showed a tendency for better adherence to hygienic procedures by medical students compared to qualified physicians. After analyzing the variables affecting the beliefs of physicians and their perception of hand hygiene, it was shown that a positive attitude to hand hygiene after contact with a patient, belief in being a role model for other colleagues, and awareness of being watched were related to them following hand washing and disinfection procedures [19].

In addition to worse hand hygiene compliance rates in the group of physicians, the principles of the BBE concept to a lesser extent was observed. Mernelius et al. demonstrated that physicians presented a significantly worse adherence to the principles of “dress code” (BBE) compared to midwives and auxiliary nurses [29]. Our results also confirm this fact; in particular, physicians were more often in the non-BBE group (53.3%) than in the BBE group (46.7%) (*p* = 0.041). It has been proven that multi-module health campaigns provide measurable benefits for the patient, medical staff, and the medical facility itself. It should be emphasized that they contribute to minimizing the number of nosocomial infections and significantly increase the rate of compliance with hand washing and disinfection procedures. “Clean Care is Safer Care”—this was the motto of the WHO campaigns conducted in the years 2005–2009, and how relevant it is at present. A multi-module hand hygiene strategy is essential to increase hand hygiene adherence and monitoring [14,30]. During the pilot campaign organized by the WHO, which lasted 3 months and was conducted in eight countries, the average rate of hand hygiene compliance increased from 39.6% to 56.9% [31].

As far as we know, this is one of the first systematic evaluations of hand disinfection techniques among medical and non-medical personnel of Polish origin from a multicenter aspect. Another strength of our study is that it is based on a relatively large population. Unfortunately, several limitations of our study also need to be addressed. Some parts of our results are based on self-reported data. However, in terms of demographic data, questionnaires are the only known implements available for large-scale population investigations so far. Moreover, only Polish participants were recruited for our study; therefore, in order to verify our findings in other national groups, the data should be replicated in other populations with different regulations and BBE politics.

## 5. Conclusions

In this study, we prove that the education of medical personnel in improving hand hygiene is necessary. Moreover, a significant positive impact of compliance with the BBE concept for effective hand disinfection was demonstrated. The chance of complying with the BBE concept increased among employees working for less than 10 years in treatment wards, operating theatres, and neonatology or rehabilitation wards. Furthermore, improvements in hand hygiene and BBE compliance were highlighted as the most effective measures to reduce the transmission of pathogenic microorganisms in healthcare facilities. This is why continued education, control, and infection prevention can be the keys to improving the BBE policy’s effectiveness.

## Figures and Tables

**Table 1:** The frequency of adherence to the BBE concept, depending on the profession.

	BBE N = 3932	Non-BBE N = 3612	Total N = 7544
Medical personnel	2712 (51.5%)	2559 (48.5%)	5271 (100.0%)
Physicians	687 (46.7%)	783 (53.3%)	1470 (100.0%)
Nurses	2025 (53.3%)	1776 (46.7%)	3801 (100.0%)
Non-medical personnel (other)	1220 (53.7%)	1053 (46.3%)	2273 (100.0%)
Cleaning service	522 (51.4%)	494 (48.6%)	1016 (100.0%)
Food service	119 (52.9%)	106 (47.1%)	225 (100.0%)
Physiotherapists	154 (67.8%)	73 (32.2%)	227 (100.0%)
Radiologic technologists	91 (47.6%)	100 (52.4%)	191 (100.0%)
Administration specialists	216 (53.3%)	189 (46.7%)	405 (100.0%)
Pharmacists	57 (52.8%)	51 (47.2%)	108 (100.0%)
Laboratory staff	61 (60.4%)	40 (39.6%)	101 (100.0%)

**Table 2:** Assessment of compliance with the BBE principle among hospital employees.

Location of Work	Physicians N = 1395	Nurses N = 3436	Medical Personnel N = 4831	Non-Medical Personnel N = 2065	Total N = 6896
Surgical departments	334/243 *	750/514	1084/757	213/154	1297/911
	57.9/42.1	59.3/40.7	58.9/41.1	58.0/42.0	58.7/41.3
Operating theatres	92/25	193/61	285/86	43/17	328/103
	78.6/21.4	76.0/24.0	76.8/23.2	71.7/28.3	76.1/23.9
Orthopedics	36/22	71/57	107/79	35/27	142/106
	62.1/37.9	55.5/44.5	57.5/42.5	56.5/43.5	57.3/42.7
Surgery	129/122	336/261	465/383	106/86	571/469
	51.4/48.6	56.3/43.7	54.8/45.2	55.2/44.8	54.9/45.1
Ophtalmology	77/74	150/135	227/209	29/24	256/233
	51.0/49.0	52.6/47.4	52.1/47.9	54.7/45.3	52.4/47.7
General departments	224/365	791/723	1015/1088	456/412	1471/1500
	38.0/62.0	52.2/47.8	48.3/51.7	52.5/47.5	49.5/50.5
Neonatology	46/34	108/59	154/93	74/39	228/132
	57.5/42.5	64.7/35.3	62.3/37.7	65.5/34.5	63.3/36.7
Pediatrics	28/55	86/58	114/113	24/21	138/134
	33.7/66.3	59.7/40.3	50.2/49.8	53.3/46.7	50.7/49.3
Internal medicine	114/212	478/482	592/694	164/224	756/918
	35.0/65.0	49.8/50.2	46.0/54.0	42.3/57.7	45.2/54.8
Neurology	22/43	52/51	74/94	23/40	97/134
	33.8/66.2	50.5/49.5	44.0/56.0	36.5/63.5	42.0/58.0
Rehabilitation	14/21	67/73	81/94	171/88	252/182
	40.0/60.0	47.9/52.1	46.3/53.7	66.0/34.0	58.1/41.9
ICU	52/39	136/125	188/164	27/20	215/184
	57.1/42.9	52.1/47.9	53.4/46.6	57.4/42.6	53.9/46.1
ED	13/19	66/108	79/127	84/101	163/228
	40.6/59.4	37.9/62.1	38.4/61.6	45.4/54.6	41.7/58.3
Other **	45/61	112/111	157/172	311/287	468/459
	42.5/57.5	50.2/49.8	47.7/52.3	52.0/48.0	50.5/49.5
Total	668/727	1855/1581	2523/2308	1091/974	3614/3282
	47.9/52.1	54.0/46.0	52.2/47.8	52.8/47.2	52.4/47.6

* BBE/non-BBE groups (number, %). ** Other = administration, radiology, laboratory, pharmacy. ICU—intensive care unit; ED—emergency department.

**Table 3:** Characteristics of personnel who adhere to the BBE concept (BBE group).

	Medical Personnel				
	Physicians N = 687	Nurses N = 2025	Total N = 2712	Other ** N = 1220	Total N = 3932
Sex					
Female	279 (40.6) *	1974 (97.5)	2253 (83.1)	1026 (84.1)	3279 (83.4)
Male	408 (59.4)	51 (3.5)	459 (16.9)	194 (15.9)	653 (16.6)
Time of participation in the study					
The first time (1x)	620 (90.2)	1843 (91.0)	2463 (90.8)	1079 (88.4)	3542 (90.1)
The second time (2x)	67 (9.8)	182 (9.0)	249 (9.2)	141 (11.6)	390 (9.9)
Job seniority					
≤10 years	438 (63.8)	1158 (57.2)	1596 (58.8)	710 (37.2)	2306 (58.6)
>10 years	249 (36.2)	867 (42.8)	1116 (41.2)	510 (41.8)	1626 (41.4)
Location of work					
Hospital	668 (97.3)	1855 (91.6)	2523 (93.0)	1091 (89.4)	3614 (91.9)
Clinic	16 (2.3)	119 (5.9)	135 (5.0)	47 (3.9)	182 (4.6)
LTCF	3 (0.4)	51 (2.5)	54 (2.0)	82 (6.7)	136 (3.5)
Hospital	668	1855	2523	1091	3614
Surgical departments	334 (50.0)	750 (40.4)	1084 (43.0)	213 (19.5)	1297 (35.9)
General departments	224 (33.5)	791 (42.6)	1015 (40.2)	456 (41.8)	1471 (40.7)
ED	13 (1.9)	66 (3.6)	79 (3.1)	84 (7.7)	163 (4.5)
ICU	52 (7.9)	136 (7.4)	188 (7.5)	27 (2.5)	215 (5.9)
Other	45 (6.7)	112 (6.0)	157 (6.2)	311 (28.5)	468 (13.0)
Level of healthcare referral system	668	1855	2523	1091	3614
Primary	316 (47.3)	1078 (58.1)	1394 (55.3)	646 (59.2)	2040 (56.4)
Secondary	174 (26.0)	435 (23.5)	609 (24.1)	290 (26.6)	899 (24.9)
Tertiary	178 (26.7)	342 (18.4)	520 (20.6)	155 (14.2)	675 (18.7)

* Number (%). ** Non-medical personnel. ICU—intensive care unit; ED—emergency department; LTCFs—long-term-care facilities.

**Table 4:** Characteristics of personnel who do not adhere to the BBE concept (non-BBE group).

	Medical Personnel				
	Physicians N = 687	Nurses N = 2025	Total N = 2712	Other ** N = 1220	Total N = 3932
Sex					
Female	424 (54.2) *	1750 (98.5)	2174 (85.0)	885 (84.0)	3059 (84.7)
Male	359 (45.8)	26 (1.5)	385 (15.0)	168 (16.0)	553 (15.3)
Time of participation in the study					
The first time (1x)	703 (89.8)	1588 (89.4)	2291 (89.5)	939 (89.2)	3230 (89.4)
The second time (2x)	80 (10.2)	188 (10.6)	268 (10.5)	114 (10.8)	382 (10.6)
Job seniority					
≤10 years	473 (60.4)	1048 (59.0)	1521 (59.4)	634 (60.2)	2155 (59.7)
>10 years	310 (39.6)	728 (41.0)	1038 (40.6)	419 (39.8)	1457 (40.3)
Location of work					
Hospital	727 (92.9)	1581 (89.0)	2308 (90.2)	974 (92.5)	3282 (90.8)
Clinic	52 (6.6)	156 (8.8)	208 (8.1)	48 (4.6)	256 (7.1)
LTCF	4 (0.5)	39 (2.2)	43 (1.7)	31 (2.9)	74 (2.1)
Hospital	727	1581	2308	974	3282
Surgical departments	243 (33.4)	514 (32.5)	757 (32.8)	154 (15.8)	911 (27.8)
General departments	224 (30.8)	723 (45.7)	947 (41.1)	412 (42.3)	1500 (45.7)
ED	19 (2.6)	108 (6.8)	127 (5.5)	101 (10.4)	228 (6.9)
ICU	39 (5.4)	125 (7.9)	164 (7.1)	20 (2.0)	184 (5.6)
Other	61 (8.4)	111 (7.0)	172 (7.5)	287 (29.5)	459 (14.0)
Level of healthcare referral system	727	1581	2308	974	3282
Primary	362 (49.8)	993 (62.8)	1355 (58.7)	573 (58.8)	1928 (58.7)
Secondary	176 (24.2)	391 (24.7)	567 (24.6)	260 (26.7)	827 (25.2)
Tertiary	189 (26.0)	197 (12.5)	386 (16.7)	141 (14.5)	527 (16.1)

* Number (%). ** Non-medical personnel. ICU—intensive care unit; ED—emergency department; LTCFs—long-term-care facilities.

**Table 5:** Assessment of the correctness of hand disinfection behavior and compliance with the BBE concept in various professional groups.

	Correct Disinfection	Incorrect Disinfection	Total
Medical personnel (5271)	2043/1462 *	669/1097	2712/2559
Physicians (1470)	511/415	176/368	687/783
Nurses (3801)	1532/1047	493/729	2025/1776
Non-medical personnel (2273)	832/542	388/511	1220/1053
Total (7544)	2875/2004	1057/1608	3932/3612

* BBE/non-BBE groups.

## Data Availability

The datasets used and/or analyzed during the current study are available from the corresponding author upon reasonable request.

## References

[1] Loveday H.P., Wilson J.A., Pratt R.J., Golsorkhi M., Tingle A., Bak A., Browne J., Prieto J., Wilcox M., UK Department of Health (2014). epic3: National evidence-based guidelines for preventing healthcare-associated infections in NHS hospitals in England. J. Hosp. Infect..

[2] Gupta M.K., Lipner S.R. (2020). Hand hygiene in preventing COVID-19 transmission. Cutis.

[3] Allegranzi B., Sax H., Pittet D. (2013). Hand hygiene and healthcare system change within multi-modal promotion: A narrative review. J. Hosp. Infect..

[4] Mathai E., Allegranzi B., Seto W.H., Chraiti M.N., Sax H., Larson E., Pittet D. (2010). Educating healthcare workers to optimal hand hygiene practices: Addressing the need. Infection.

[5] Cherry M.G., Brown J.M., Bethell G.S., Neal T., Shaw N.J. (2012). Features of educational interventions that lead to compliance with hand hygiene in healthcare professionals within a hospital care setting. A BEME systematic review: BEME Guide No. 22. Med. Teach..

[6] Huis A., Schoonhoven L., Grol R., Donders R., Hulscher M., van Achterberg T. (2013). Impact of a team and leaders-directed strategy to improve nurses’ adherence to hand hygiene guidelines: A cluster randomised trial. Int. J. Nurs. Stud..

[7] Martos-Cabrera M.B., Mota-Romero E., Martos-Garcia R., Gomez-Urquiza J.L., Suleiman-Martos N., Albendin-Garcia L., Canadas-De la Fuente G.A. (2019). Hand Hygiene Teaching Strategies among Nursing Staff: A Systematic Review. Int. J. Environ. Res. Public Health.

[8] Lotfinejad N., Peters A., Tartari E., Fankhauser-Rodriguez C., Pires D., Pittet D. (2021). Hand hygiene in health care: 20 years of ongoing advances and perspectives. Lancet Infect. Dis..

[9] Vermeil T., Peters A., Kilpatrick C., Pires D., Allegranzi B., Pittet D. (2019). Hand hygiene in hospitals: Anatomy of a revolution. J. Hosp. Infect..

[10] Gould D.J., Moralejo D., Drey N., Chudleigh J.H., Taljaard M. (2017). Interventions to improve hand hygiene compliance in patient care. Cochrane Database Syst. Rev..

[11] Luangasanatip N., Hongsuwan M., Limmathurotsakul D., Lubell Y., Lee A.S., Harbarth S., Day N.P., Graves N., Cooper B.S. (2015). Comparative efficacy of interventions to promote hand hygiene in hospital: Systematic review and network meta-analysis. BMJ.

[12] Erasmus V., Daha T.J., Brug H., Richardus J.H., Behrendt M.D., Vos M.C., van Beeck E.F. (2010). Systematic review of studies on compliance with hand hygiene guidelines in hospital care. Infect. Control Hosp. Epidemiol..

[13] Szumska E., Czajkowski P., Zablocki M., Rozkiewicz D. (2022). The Association between Hand Disinfection Techniques and Their Barriers, as Well as the “Bare below the Elbows” Concept, among Healthcare Professionals—A Study Based on a Polish Population. Int. J. Environ. Res. Public Health.

[14] (2009). WHO Guidelines on Hand Hygiene in Health Care: First Global Patient Safety Challenge Clean Care Is Safer Care.

[15] Al-Allak A., Sarasin S., Key S., Morris-Stiff G. (2008). Wedding rings are not a significant source of bacterial contamination following surgical scrubbing. Ann. R. Coll. Surg. Engl..

[16] Burger A., Wijewardena C., Clayson S., Greatorex R.A. (2011). Bare below elbows: Does this policy affect handwashing efficacy and reduce bacterial colonisation?. Ann. R. Coll. Surg. Engl..

[17] Willis-Owen C.A., Subramanian P., Kumari P., Houlihan-Burne D. (2010). Effects of ‘bare below the elbows’ policy on hand contamination of 92 hospital doctors in a district general hospital. J. Hosp. Infect..

[18] Boyce J.M., Pittet D., Healthcare Infection Control Practices Advisory Committee, HICPAC/SHEA/APIC/IDSA Hand Hygiene Task Force (2002). Guideline for Hand Hygiene in Health-Care Settings. Recommendations of the Healthcare Infection Control Practices Advisory Committee and the HIPAC/SHEA/APIC/IDSA Hand Hygiene Task Force. Am. J. Infect. Control.

[19] Pittet D., Simon A., Hugonnet S., Pessoa-Silva C.L., Sauvan V., Perneger T.V. (2004). Hand hygiene among physicians: Performance, beliefs, and perceptions. Ann. Intern. Med..

[20] Smiddy M.P., O’ Connell R., Creedon S.A. (2015). Systematic qualitative literature review of health care workers’ compliance with hand hygiene guidelines. Am. J. Infect. Control.

[21] Fonguh S., Uwineza A., Catry B., Simon A. (2016). Belgian hand hygiene campaigns in ICU, 2005–2015. Arch. Public Health.

[22] Lee S.S., Park S.J., Chung M.J., Lee J.H., Kang H.J., Lee J.A., Kim Y.K. (2014). Improved Hand Hygiene Compliance is Associated with the Change of Perception toward Hand Hygiene among Medical Personnel. Infect. Chemother..

[23] McLaws M.L., Farahangiz S., Palenik C.J., Askarian M. (2015). Iranian healthcare workers’ perspective on hand hygiene: A qualitative study. J. Infect. Public Health.

[24] Mortell M., Balkhy H.H., Tannous E.B., Jong M.T. (2013). Physician ‘defiance’ towards hand hygiene compliance: Is there a theory-practice-ethics gap?. J. Saudi Heart Assoc..

[25] Hautemaniere A., Cunat L., Diguio N., Vernier N., Schall C., Daval M.C., Ambrogi V., Tousseul S., Hunter P.R., Hartemann P. (2010). Factors determining poor practice in alcoholic gel hand rub technique in hospital workers. J. Infect. Public Health.

[26] Skodova M., Garcia Urra F., Gimeno Benitez A., Jimenez Romano M.R., Gimeno Ortiz A. (2015). Hand hygiene assessment in the workplace using a UV lamp. Am. J. Infect. Control.

[27] Randle J., Arthur A., Vaughan N. (2010). Twenty-four-hour observational study of hospital hand hygiene compliance. J. Hosp. Infect..

[28] Pittet D., Mourouga P., Perneger T.V. (1999). Compliance with handwashing in a teaching hospital. Infection Control Program. Ann. Intern. Med..

[29] Mernelius S., Svensson P.O., Rensfeldt G., Davidsson E., Isaksson B., Lofgren S., Matussek A. (2013). Compliance with hygiene guidelines: The effect of a multimodal hygiene intervention and validation of direct observations. Am. J. Infect. Control.

[30] Pittet D., Donaldson L. (2005). Clean care is safer care: The first global challenge of the WHO World Alliance for Patient Safety. Am. J. Infect. Control.

[31] Allegranzi B., Gayet-Ageron A., Damani N., Bengaly L., McLaws M.L., Moro M.L., Memish Z., Urroz O., Richet H., Storr J. (2013). Global implementation of WHO’s multimodal strategy for improvement of hand hygiene: A quasi-experimental study. Lancet Infect. Dis..

